# Performance Assessment of μTASWako i50, a New Microfluidic Immunoassay System for Hepatocellular Carcinoma Biomarkers AFP, AFP-L3%, and PIVKA-II

**DOI:** 10.7759/cureus.103793

**Published:** 2026-02-17

**Authors:** Tomoyasu Yoshikawa, Takuma Ohtsubo, Isao Yamaguchi, Hiroko Nishihara, Yasuhiro Mori, Hiroyuki Yamada, Hidenori Toyoda, Tomohisa Kawabata, Shinjiro Matsuda

**Affiliations:** 1 Medical Systems Research and Development Center, FUJIFILM Corporation, Amagasaki, JPN; 2 Diagnostics Technical Service and Department Operations, FUJIFILM Wako Pure Chemical Corporation, Osaka, JPN; 3 Medical Systems Business Division, FUJIFILM Corporation, Tokyo, JPN; 4 Department of Gastroenterology, Ogaki Municipal Hospital, Ogaki, JPN

**Keywords:** afp and pivka-ii, clinical application, glycosylation, hepatocellular carcinoma (hcc), lectin, micro total analysis system, separation analysis, serum biomarkers

## Abstract

Introduction

Microfluidic Total Analysis Systems (µTAS) miniaturize and automate immunoassay procedures by integrating reaction and electrophoretic separation within microchannels, enabling the concentration and separation of immunocomplexes. Since 2009, the µTASWako i30 system has been used to measure biomarkers of hepatocellular carcinoma (HCC), such as AFP, AFP-L3%, and PIVKA-II, using a fluorescence immunoassay based on the liquid-phase binding assay-electrokinetic analyte transport assay method combined with isotachophoresis (ITP) and capillary gel electrophoresis (CGE). This technique enables simultaneous quantitative and qualitative analysis of glycosylation variants exemplified by AFP-L3. The µTASWako i30 system, however, has limitations in throughput and measurement range for clinical samples with high biomarker concentrations. The µTASWako i50 system, a successor device, introduces modifications such as increased measurement speed and automatic sample dilution to address these issues. This study aims to evaluate the analytical performance of the µTASWako i50 system compared with the µTASWako i30.

Methods

The µTASWako i50 system retains the microfluidic immunoassay principles of the i30. Sequential operations of dispensing, immune complex formation, ITP stacking, and CGE with laser-induced fluorescence detection are performed on disposable plastic chips. Modifications include accelerated processing and an automated dilution function for samples exceeding assay linearity. Analytical parameters such as sensitivity, reproducibility, linearity, and correlation with the i30 system were assessed using an appropriate combination of standard solutions, control materials, and clinical serum samples from patients with chronic liver diseases, including hepatitis, cirrhosis, and HCC. Thereby, traceability of measurement values to the predecessor system was also examined.

Results

Analytical performance of the µTASWako i50 was found to be comparable to that of the i30 system with respect to sensitivity, reproducibility, and linearity across the tested biomarkers. The automated dilution function extended the measurement range, enabling quantitative analysis of samples with elevated biomarker levels without manual dilution. Correlation of measurement values between the two systems showed high agreement, and assay throughput was increased while turnaround time was reduced under the tested conditions using the µTASWako i50 system.

Conclusions

The use of the µTASWako i50 should enhance laboratory workflows by increasing processing efficiency and minimizing manual handling. The system provides reliable analytical performance comparable to its predecessor, thereby supporting consistent longitudinal clinical measurements of key biomarkers. Furthermore, the µTASWako i50 has the potential to improve clinical utility through an expanded measurement range and operational automation and may facilitate ongoing research into cancer biomarkers characterized by alterations in glycosylation patterns.

## Introduction

The concept of microfluidic Total Analysis Systems (µTAS) was first introduced in 1990 by Manz et al. [1] as a novel approach to miniaturizing and automating chemical analysis using microchannels, laying the foundation for µTAS as a transformative technology in analytical chemistry. In 1992, Harrison et al. [2] demonstrated the feasibility of fabricating functional µTAS devices using micromachining techniques to create microchannels on glass chips. Both studies noted the potential of µTAS for applications in clinical diagnostics, among other fields, emphasizing its ability to simplify and automate complex analytical processes. After years of development [3,4], the µTASWako i30 system, one of the first microfluidic systems approved for clinical diagnostic testing, was introduced in Japan in 2009 and in the United States in 2011. The µTASWako i30 performs immunoassays utilizing electrophoresis in microchannels molded in plastic chips to separate and quantitate analytes in serum samples.

The µTASWako i30 system uses the liquid-phase binding assay-electrokinetic analyte transport assay method, which forms an immune complex between an antigen and a fluorescently labeled antibody while electrokinetically moving a DNA-labeled second antibody into contact with the antigen and concentrating the resulting ternary immune complex by isotachophoresis (ITP) [5]. The binding of the DNA-labeled antibody to the antigen/labeled-antibody complex forms a sandwich immune complex with high electrophoretic mobility that can be stacked and concentrated by ITP, separated by capillary gel electrophoresis (CGE), and measured by laser-induced fluorescence (LIF). To further enhance assay sensitivity and precision, this method incorporates an on-chip voltage-switching technique, which seamlessly transitions from ITP stacking to CGE separation within the microfluidic channel. This transition minimizes sample dilution and band broadening, allowing highly efficient immune complex concentration and separation [6]. The advantages of this method are that (1) reagents and samples are metered using hydrodynamic laminar flow in precision-molded microchannels on disposable plastic chips, improving assay precision and eliminating sample carryover; (2) the reaction kinetics of immune complex formation are accelerated due to reaction in the liquid phase and concentration of the reactants and immune complex by ITP stacking; (3) bound/free separation is performed simultaneously with the formation and stacking of the immune complex, thereby eliminating washing steps; and (4) detection sensitivity is greatly increased by ITP stacking of the immune complex into a highly concentrated zone prior to electrophoretic separation and LIF detection. Together, these features provide a rapid, highly sensitive, and reproducible on-chip immunoassay.

An additional feature is achieved by adding a substance with affinity for biomolecules to the separation gel and performing affinity electrophoresis, a versatile technique that enables detection and characterization of biomolecules by leveraging specific interactions between analytes and affinity ligands, as reviewed by Kinoshita et al. [7]. In this way, critical modifications in the glycan structure of glycoprotein analytes can be detected and measured using a plant lectin in the gel. This assay feature distinguishes it from traditional solid-phase immunoassay methods in that it can simultaneously quantitate and evaluate qualitative differences in the target antigen.

The µTASWako i30 system, leveraging these capabilities, facilitated the development of reagents targeted at the simultaneous measurement of total AFP and AFP-L3% [8]. The AFP isoform, AFP-L3, can be measured using *Lens culinaris* agglutinin (LCA) in the separation gel, which binds to the terminal fucosyl residue present only on the carbohydrate chain of the AFP-L3 isoform, thereby separating it from AFP lacking the fucosyl residue during the CGE step of the assay. The L3 isoform has been shown to be a biomarker for hepatocellular carcinoma (HCC) [9], and in combination with µTASWako reagents for PIVKA-II (des-gamma carboxyprothrombin or DCP), these assays can be used to monitor high-risk patients for HCC [10]. Later, an immunoassay for procalcitonin, a sepsis biomarker that requires rapid and sensitive test results, was also released, and together these reagents and the system have contributed valuable new assays to the clinical diagnostic field.

AFP, AFP-L3%, and PIVKA-II are currently widely recognized as important biomarkers for HCC and are used in clinical practice for diagnostic purposes and risk stratification. While commonly reported cutoff values, such as AFP levels of 20 ng/mL, AFP-L3% levels of approximately 10%, and PIVKA-II levels of 40 mAU/mL, are frequently used in clinical studies, these values are not universally standardized for surveillance and should be interpreted in the context of individual patient risk factors and clinical settings. The clinical practice guidelines developed by the Japan Society of Hepatology recommend these markers as adjuncts to imaging modalities such as ultrasonography for HCC surveillance. While international guidelines, such as those from EASL and AASLD, do not recommend routine use of blood-based biomarkers for HCC surveillance, recent research has explored the integration of these markers into advanced algorithms such as the GALAD model, which combines gender, age, AFP, AFP-L3%, and PIVKA-II to enhance the performance of HCC surveillance and early detection [10,11].

Despite its success in clinical diagnostics, the µTASWako i30 system has encountered limitations in two key areas: sample throughput and dynamic measurement range. The system’s processing speed can become a bottleneck when managing high volumes of samples, and its dynamic measurement range is insufficient for patients with significantly elevated AFP or PIVKA-II levels, requiring manual dilution steps. Addressing these challenges is critical for improving laboratory efficiency and ensuring accurate biomarker quantification across a wide range of concentrations. These enhancements are particularly important for increasing the system’s clinical utility and ease of use in diverse diagnostic scenarios.

To respond to these needs, we developed a next-generation µTASWako i50 system incorporating key improvements such as faster processing speeds, an expanded measurement range, and automated sample dilution capabilities. These innovations aim to ensure accurate biomarker quantification in high-concentration samples while reducing manual intervention and associated errors. The primary objective of this study is to evaluate the analytical performance of the µTASWako i50 system, including its sensitivity, reproducibility, and clinical applicability for biomarker detection. Secondary objectives include assessing the impact of automated sample dilution, improved throughput, and implementation of the warning region feature for AFP-L2 detection. These advancements are expected to address the limitations of the µTASWako i30 system and enhance the clinical utility of the µTASWako i50 system in diverse diagnostic scenarios.

## Materials and methods

µTASWako i50 microfluidic chip

**Figure 1:**
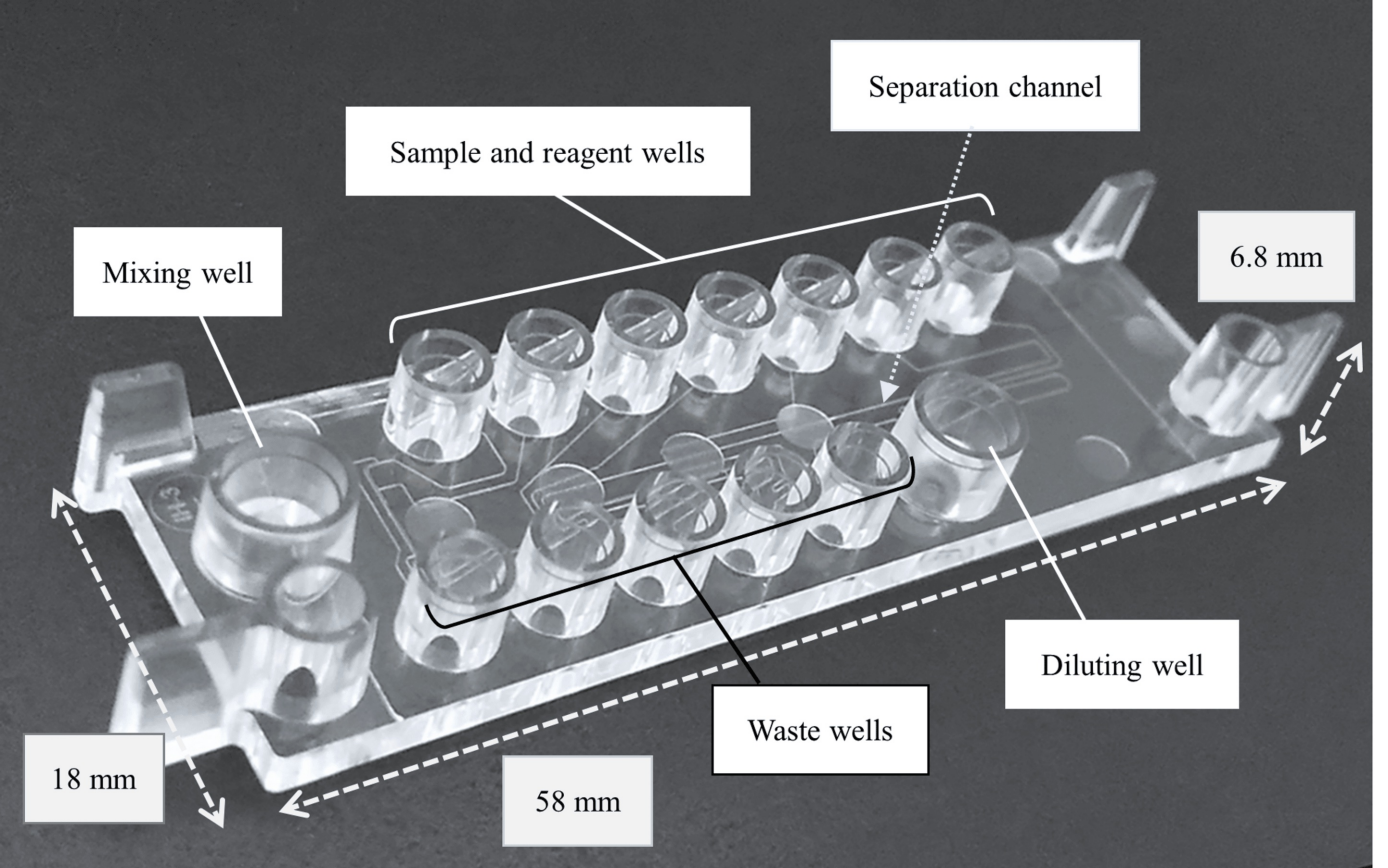
Microfluidic chip for µTASWako i50 Image credit: Tomoyasu Yoshikawa

The microfluidic chip (Figure 1) was manufactured by injection molding polymethyl methacrylate (PMMA) resin to form precision-molded channels connecting the Reagent, Sample, and Waste wells. The channels were sealed by laminating the chip with a PMMA film. In addition to the reagent, sample, and waste wells for dispensing solutions into the channels and applying differential pressure for solution loading, there are also wells not connected to the channels for mixing the sample buffer (SB), fluorescently labeled antibody, and sample (mixing well). A new well for diluting the sample with dilution buffer (diluting well) was added to the µTASWako i50 chip, allowing automatic sample dilution when requested by the operator. The addition of the diluting well makes the i50 chip slightly larger than the i30 chip.

**Figure 2:**
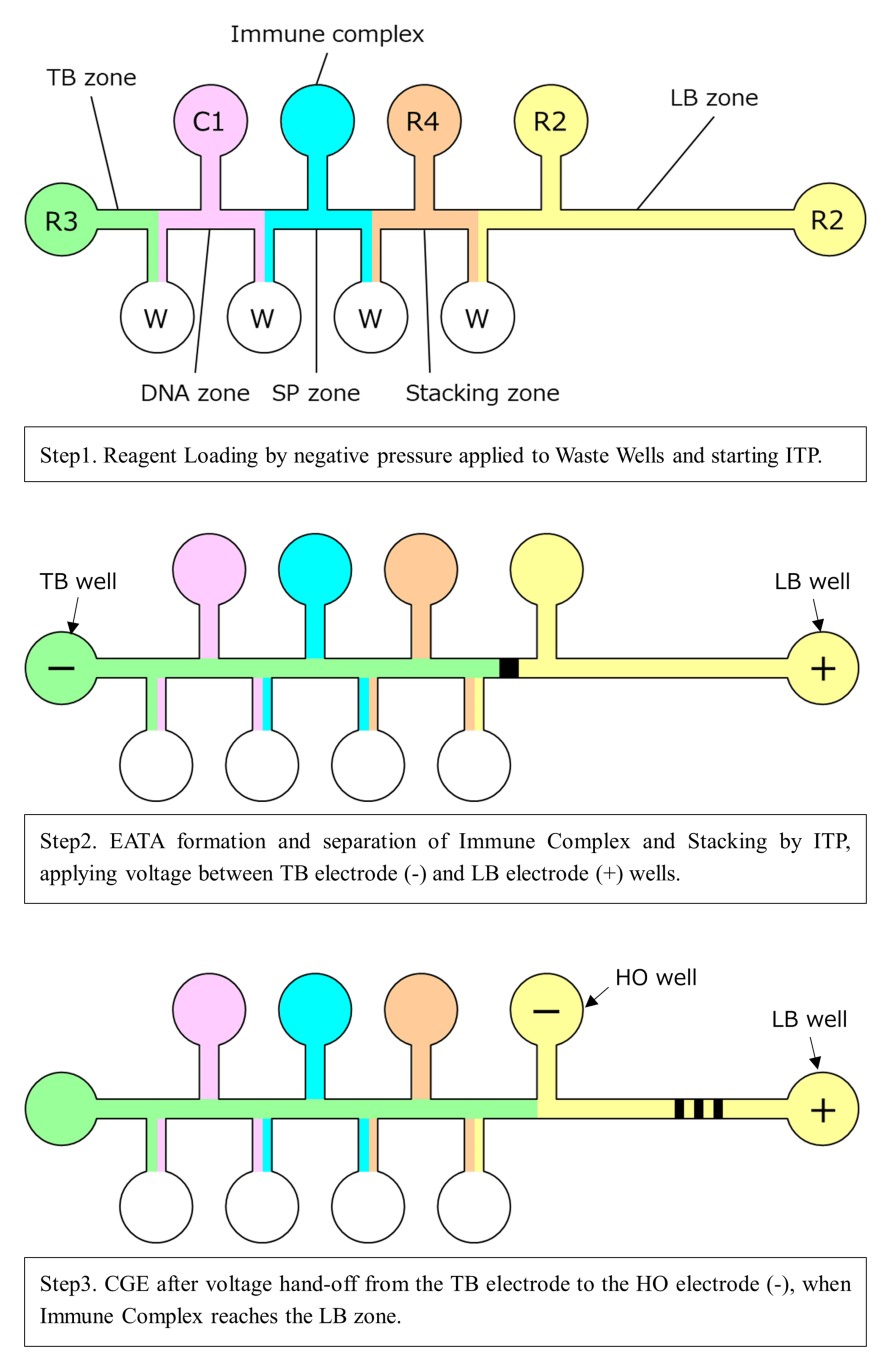
Zones in the channel and three steps of ITP-CGE CGE, capillary gel electrophoresis; EATA, electrokinetic analyte transport assay; HO, hand-off; ITP, isotachophoresis; LB, leading buffer; R, reagent; SP, sample preparation; TB, trailing buffer; W, waste Image credit: Tomoyasu Yoshikawa

Within the channel, the following zones are connected in series (Figure 2): the TB zone, filled with “trailing buffer” (TB; electrophoresis buffer 3: R3) containing HEPES trailing ions; the DNA zone, filled with DNA-labeled antibody in “labeled antibody solution” (C1); the SP zone, filled with fluorescent dye-labeled antibody and sample diluted with SB (electrophoresis buffer 1: R1); the ST zone, filled with “stacking buffer” (ST; electrophoresis buffer 4: R4); and the LB zone, filled with “leading buffer” (LB; electrophoresis buffer 2: R2) containing chloride as the leading ion.

The TB plays an important role in ITP by providing a stable environment as the analytes (referring to immune complexes) move through the channel. The trailing ions, with lower electrophoretic mobility, stabilize the rear boundary of the sample zones, ensuring that the analytes remain in a concentrated zone during the electrophoretic process. The SB provides a medium for the fluorescent dye-labeled antibodies to interact with the antigen. By optimizing the reagent composition of the SB, the antigen-antibody reaction is facilitated and nonspecific reactions with the serum sample matrix are reduced, thereby enhancing detection sensitivity during subsequent analysis. The ST promotes sufficient sample stacking to concentrate the analytes into a narrow band by ITP, forming a high-voltage gradient between the trailing ion (HEPES) and leading ion (chloride), with analytes of intermediate mobility stacked between them. This creates a sharp interface between the analytes and the buffers, enhancing the resolution of the separation process. The ST zone can also be used as a second SP zone in the PIVKA-II assay to increase sample volume and assay sensitivity.

The LB, together with a separation gel used in this zone, facilitates separation of the immune complex. Like the TB, the LB plays a key role in ITP by ensuring that analytes are effectively stacked and separated. It maintains a sharp interface between the analytes and the buffers, further enhancing the resolution of the separation process.

In AFP-L3% measurement, lentil lectin, LCA, is added to the LB, and fucosylated AFP (AFP-L3), which has affinity for LCA, is separated by affinity electrophoresis from the unfucosylated fraction (AFP-L1) and measured. The channel width of the LB zone has been reduced to 40 µm, which is half that of the microfluidic chip for µTASWako i30, allowing higher voltage to be applied during separation to shorten assay time and increase test throughput without excessively increasing temperature due to Joule heating. By enabling higher voltage and improved thermal management, the system can maintain performance stability under high-throughput conditions. This is particularly important in clinical settings where timely diagnosis can critically impact patient outcomes.

**Figure 3:**
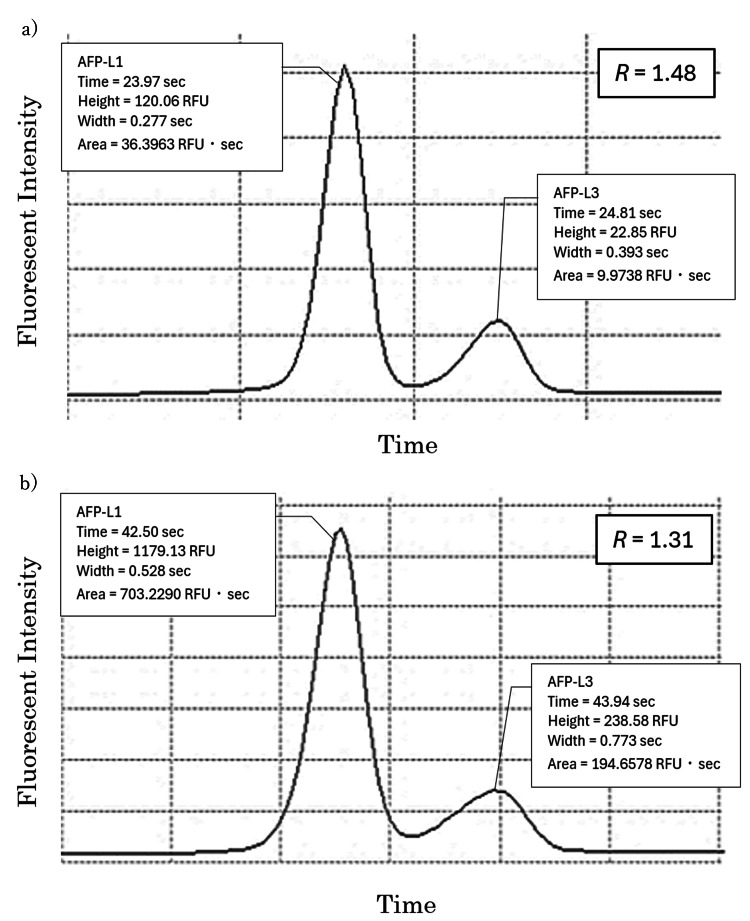
Electropherogram obtained when measuring a control sample with (a) µTASWako i50 and (b) µTASWako i30

In addition to faster separation, the narrower channel width in CGE increases assay sensitivity and reproducibility by reducing band broadening, producing sharper peaks, and enabling consistent detection of low-abundance analytes. It also improves separation accuracy of similar analytes, such as AFP isoforms (AFP-L1 and AFP-L3), which is crucial for reliable biomarker quantification and diagnosis. As illustrated in Figure 3, peak separation between AFP-L1 and AFP-L3 was evaluated by determining resolution (R) for the i50 and i30 chips.

R indicates the degree of separation from adjacent peaks and is calculated using the following formula:

$R = 1.18 \times \frac{\text{L3 Time} - \text{L1 Time}}{\text{L1 Width} + \text{L3 Width}}$

A larger R indicates better separation of adjacent peaks using the narrower-channel i50 chip.

Furthermore, the redesigned chip architecture promotes the possibility of reducing reagent consumption, as narrower channels require less reagent for filling. This aspect of the design holds potential for future optimization, which could lead to lower operational costs and increased assay efficiency. Together, these enhancements demonstrate significant advancements of the µTASWako i50 system over the i30, providing a robust and efficient solution for contemporary clinical diagnostics.

µTASWako i50 instrument

**Figure 4:**
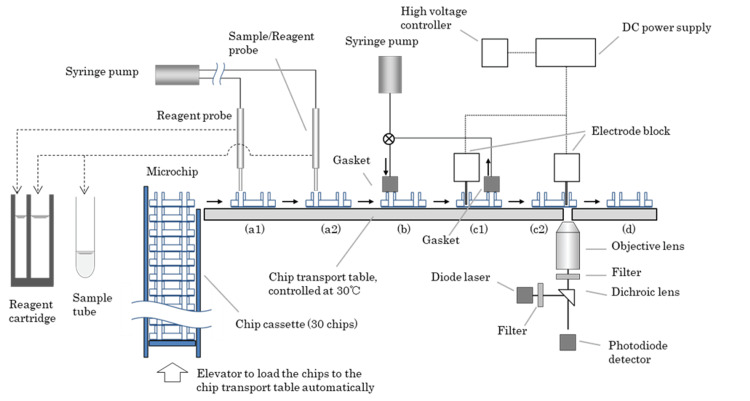
µTASWako i50 system configuration Image credit: Takuma Ohtsubo

The system configuration of the instrument is shown in Figure 4. Thirty microfluidic chips are stored in each chip cassette, and five chip cassettes (150 chips total) can be installed in the instrument, allowing chips to be replenished during sample testing. Up to eight reagent cartridges can be loaded into the instrument’s refrigerated storage. Each cartridge is prefilled with reagents for 100 tests, as described in the µTASWako i50 Reagents section. Specimens are placed in dedicated sample racks (10 samples per rack), which can be installed in six sample rack slots in any order and at any time during the test run. Both barcoded sample tubes and sample cups of approximately 100 µL can be used, and measurements can be performed with a minimum of 35 µL using a dedicated trace sample rack.

When a test is initiated, a microfluidic chip is automatically removed from the cassette and placed onto the chip carrier in the instrument. The chip moves along the transport surface to dispensing position a1, where the dispensing probe delivers reagents into the predetermined wells (Figure 2, Step 1). The chip then moves to position a2, where the sample and remaining reagents are dispensed. The filled chip proceeds to the priming section (b), where reagents are introduced into the channel by briefly applying positive pressure (138 kPa) supplied from a syringe pump through a manifold pressed against the chip wells. Prior to initiating electrophoresis at the first electrophoresis position (c1), negative pressure (-35 kPa) is applied to the waste wells for a short time to sharpen the liquid interfaces between zones due to laminar flow from the reagent and sample wells to the waste wells.

ITP is initiated by applying a voltage between the TB well and the LB well (Figure 2, Step 2). The DNA-labeled antibody is concentrated and transported by ITP to the SP zone, where it forms a ternary complex with the analyte and fluorescent dye-labeled antibody. ITP transport through the SP zone also separates the immune complex from unbound fluorescently labeled antibody, which has lower electrophoretic mobility and is not stacked during ITP. The immune complex migrates toward the anode while being further concentrated by ITP stacking. Once it advances into the ST zone, the voltage is stopped, and the chip moves to the second electrophoresis section (c2).

After ITP is resumed, the instrument continuously monitors the voltage ratio in the channel between the TB, LB, and Handoff (HO) electrodes. When a voltage ratio fluctuation is detected, indicating that the boundary containing the stacked complex has reached the intersection of the HO channel and LB zone (Figure 2, Step 3), the instrument switches to separation mode using CGE by switching the cathode voltage from the TB well to the HO well, while the anode voltage remains at the LB well. In separation mode, the immune complex is further separated from unwanted fluorescent noise components that were stacked with the immune complex during ITP. This on-chip ITP-CGE voltage-switching process was described in detail by Park et al. [6].

In the AFP-L3% measurement, AFP-L3 is separated from AFP-L1 because the gel contains a lectin that has an affinity for AFP-L3, thereby slowing its migration during CGE. The ternary immune complex containing the fluorescently labeled antibody is detected by LIF using the optical detection unit (excitation/emission wavelength = 640/670 nm). After the measurement is completed, the chip is moved to position d and then discarded. The instrument, designed for automated high-throughput operations, measures 690 mm in height, 700 mm in width, and 650 mm in depth, and weighs 135 kg.

µTASWako i50 reagents

Each reagent cartridge contains eight compartments, and the required amount of reagent for 100 tests is contained in each compartment. For the AFP assay, including AFP-L3%, two monoclonal mouse antibodies (clones WA1 and WA2) are used to form a sandwich immune complex. These antibodies recognize distinct epitopes on the AFP molecule and are labeled with either a 245 bp double-stranded DNA or a fluorescent dye (HiLyte Fluor 647; AnaSpec, San Jose, CA, USA) using established protocols [8]. Similarly, the PIVKA-II assay utilizes monoclonal antibodies and the same labeling chemistry, based on the same analytical principle as the AFP assay. However, the specific antibody clone identities are proprietary to the manufacturer and are not publicly disclosed. In addition to the labeled antibodies, the assay reagents include buffer components, leading ion, trailing ion, separation gel, and nonionic detergents.

The buffer compositions used in the µTASWako i50 system are as follows. For the AFP assay, the LB for ITP and CGE contains 0.2% (w/v) poly-2-dimethylaminoethyl methacrylate (pDMA), 3.4% (w/v) glycerol, 80 mM Tris-HCl (pH 7.6), 80 mM NaCl, 0.01% bovine serum albumin (BSA), and 4 mg/mL LCA. For PIVKA-II assay, the LB composition is similar but does not include LCA. The SB is composed of 3.4% (w/v) glycerol, 80 mM Tris-HCl (pH 8.0), 53 mM NaCl, 0.01% BSA, and 3.7 mM MES. The TB consists of 0.6% (w/v) pDMA, 3.4% (w/v) glycerol, 80 mM Tris, 0.01% BSA, and 138 mM HEPES. Finally, the ST comprises 3.4% (w/v) glycerol, 80 mM Tris-HCl (pH 7.5), 80 mM NaCl, and 0.01% BSA. These buffers are optimized to ensure efficient isotachophoretic stacking, separation, and detection of the immune complexes.

The overall assay format and detection principle are identical between the AFP and PIVKA-II assays, both employing the same immunoassay configuration and antibody type. While minor analyte-specific differences exist in buffer composition, the core assay architecture remains consistent. Both systems are commercially available in vitro diagnostic platforms, and all measurements were performed strictly according to the manufacturer’s instructions without modification.

The reagent’s unopened shelf life is 12 months. Once opened, the reagent can be used for up to 60 days when stored in the refrigerated storage of the µTASWako i50 instrument.

µTASWako i50 system improvements

To improve the processing throughput achieved by the µTASWako i30 system, the µTASWako i50 system increased the number of dispensing probes to two: one dedicated to reagent dispensing and the other to sample and reagent dispensing. This configuration divides the dispensing process into two steps. The electrophoresis step was also divided into two stages. The first stage consists of the ITP process, resulting in the formation of the immune complex. The second stage involves ITP to complete sample stacking, followed by CGE and LIF detection.

With two steps each for dispensing and electrophoresis, the µTASWako i50 system reduces the time required for each step (the Takt time) to approximately half that of the µTASWako i30 system. In addition, a redesigned microfluidic chip allows application of a higher CGE voltage, shortening the time required for the second electrophoresis stage. As a result, the µTASWako i50 system can complete twice as many tests as the µTASWako i30 system. Table 1 shows that reducing the takt time from 144 seconds to 72 seconds increased the test throughput from 24 to 50 measurements per hour.

**Table 1:** Throughput capacity (AFP/AFP-L3%) ^*^ Without automatic sample dilution

Parameter	μTASWako i30	μTASWako i50
Number of reagents	7	7*
Dispensing probes	1	2
Assay steps	3	5
Operations	Dispensing	Dispensing 1
	-	Dispensing 2
	Channel filling	Channel filling
	Electrophoresis	Electrophoresis 1
	-	Electrophoresis 2
Takt time for each step	144 seconds	72^*^ seconds
Throughput for one hour	24 test/h	50^*^ test/h
Time to output initial results	9 minutes	7 minutes

The µTASWako i50 system also includes a function that enables reloading of the microfluidic chip cassette during measurement. In addition, two bottles of cleaning solution for the dispensing probes are installed, significantly increasing the number of continuous measurements that can be performed without interruption (µTASWako i30: 80 measurements; µTASWako i50: 300 measurements).

To further improve convenience for measuring high-concentration samples, the device software is equipped with an automatic sample dilution function, enabling samples to be diluted automatically at the requested dilution ratio. Automated sample dilution expands the instrument’s measurable concentration range. The upper limit of the measurement range was extended by adjusting the relationship between signal intensity and light-receiving efficiency in the optical system.

Additionally, the µTASWako i50 system has an increased range of motion for the sample probe and a six-lane slot system for sample racks (compared with one lane for the µTASWako i30). Since processed sample racks are ejected from each slot to the front, the holding time of sample racks by the instrument is reduced. On the µTASWako i30 system, sample racks cannot be removed until the measurement batch is complete. This design allows samples to be smoothly transferred from the µTASWako i50 to other devices before testing runs are finished.

System calibration and sample measurement

To quantify target analytes in samples, the instrument measures a standard solution with a known analyte concentration and calculates the slope of the standard curve of analyte concentration versus the measured peak area of the immune complex. Calibration assumes a linear relationship between analyte concentration and immune complex peak area within the measurement range. The standard curve is generated using two points: a blank sample (zero concentration) and a single standard solution with a known analyte concentration. After calibration, the instrument uses the analyte peak area obtained from samples to automatically calculate measured concentrations of total AFP, AFP-L3%, and PIVKA-II. AFP-L3% is calculated as (AFP-L3 concentration / total AFP concentration) × 100.

The performance of the µTASWako i50 was evaluated by testing assay linearity, detection sensitivity (limit of detection, LOD), reproducibility (intra-assay CV), and correlation with the reference method (µTASWako i30) using clinical specimens from Ogaki Municipal Hospital. Linearity was assessed by preparing serial dilutions of analyte-rich samples with analyte-free buffer. Measured concentrations of diluted samples were plotted against expected values based on dilution factors across the intended measurement range for AFP, AFP-L3%, and PIVKA-II. The accuracy of the dilution process was confirmed by recovery rates.

Detection sensitivity was characterized by determining the LOD and limit of quantitation (LOQ). LOD was defined as the lowest analyte concentration at which the mean measured value ± two SDs did not overlap with the zero-analyte control. LOQ was defined as the lowest concentration at which the coefficient of variation (CV) was below 5% for AFP-L1 and AFP-L3 and below 10% for PIVKA-II. For these assessments, samples containing AFP-L1, AFP-L3, or PIVKA-II were serially diluted in analyte-free buffer and measured in 21 replicates per concentration.

Intra-assay precision was evaluated by measuring clinical or control samples in 10 replicates across relevant analyte concentrations. CV, calculated as the SD divided by the mean and expressed as a percentage, quantified the repeatability of the assay. Lower CV values indicate higher precision, which is essential for reliable diagnostic tests.

A method comparison between the µTASWako i50 and µTASWako i30 systems was performed using clinical serum samples. Pearson’s correlation coefficient (r) was calculated to assess the linear association between measurements, and agreement between methods was evaluated using clinically established cutoffs for each analyte. All statistical analyses were conducted using JMP^®^ 17.0.0 software (SAS Institute Inc., Cary, NC, USA), with significance set at p < 0.05.

Clinical specimens

Residual human serum samples were collected from patients at Ogaki Municipal Hospital for method comparison of the µTASWako i30 and i50 systems. For AFP and AFP-L3% assays, 155 samples were analyzed, including 73 HCC cases and 82 non-HCC cases. The median age of this cohort was 74 years (range: 34-92 years). Among the non-HCC group, 26 patients had liver cirrhosis and 56 had chronic liver disease. For PIVKA-II assays, 153 samples were evaluated, comprising 71 HCC cases and 82 non-HCC cases, with a median age of 71 years (range: 34-92 years). Non-HCC samples included 24 patients with liver cirrhosis and 58 with chronic liver disease. All samples were anonymized and stored at -80°C until analysis.

Ethical considerations

This retrospective evaluation used stored serum samples after routine clinical testing, with opt-out consent for secondary use obtained from patients. The study protocol received approval from the Life Science Ethics Committee of FUJIFILM Wako Pure Chemical Corporation (approval #RIN077, dated February 3, 2022). Written informed consent was waived due to the use of de-identified specimens.

## Results

Assay linearity

AFP/AFP-L3%

**Figure 5:**
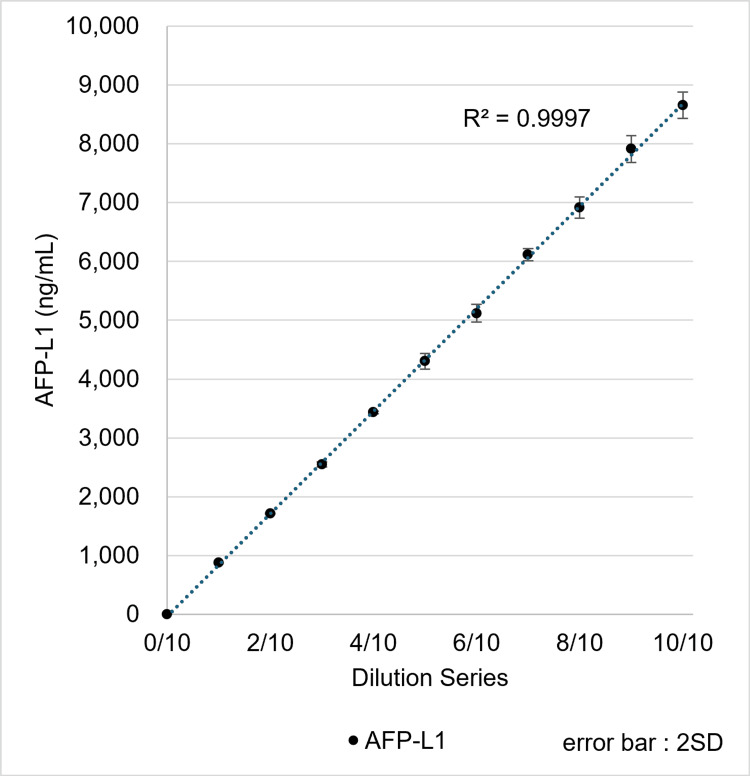
Performance for the linearity of AFP-L1

PBS containing approximately 8,600 ng/mL AFP-L1 was serially diluted with AFP-free PBS supplemented with 1% BSA, and the diluted samples were measured using the µTASWako i50. The diluent was selected to provide a consistent, well-characterized matrix and to minimize protein adsorption during measurements. AFP-L1 measurements demonstrated good linearity with respect to the dilution ratio (Figure 5). Comparable linearity was confirmed for AFP-L3 in similar experiments; these data are not shown to avoid redundancy. Based on these results, the measurable range of AFP could be extended to 8,000 ng/mL. For comparison, the US FDA-approved range for the µTASWako i30 system is up to 1,000 ng/mL, and the Japan FDA-approved range is 2,000 ng/mL. Using the automatic dilution function, dilutions of 1:4 and 1:10 were validated, yielding 101% and 104% of the expected value for a 10,075 ng/mL sample. By utilizing these automated dilutions, AFP concentrations up to 80,000 ng/mL can be measured using the µTASWako i50.

**Figure 6:**
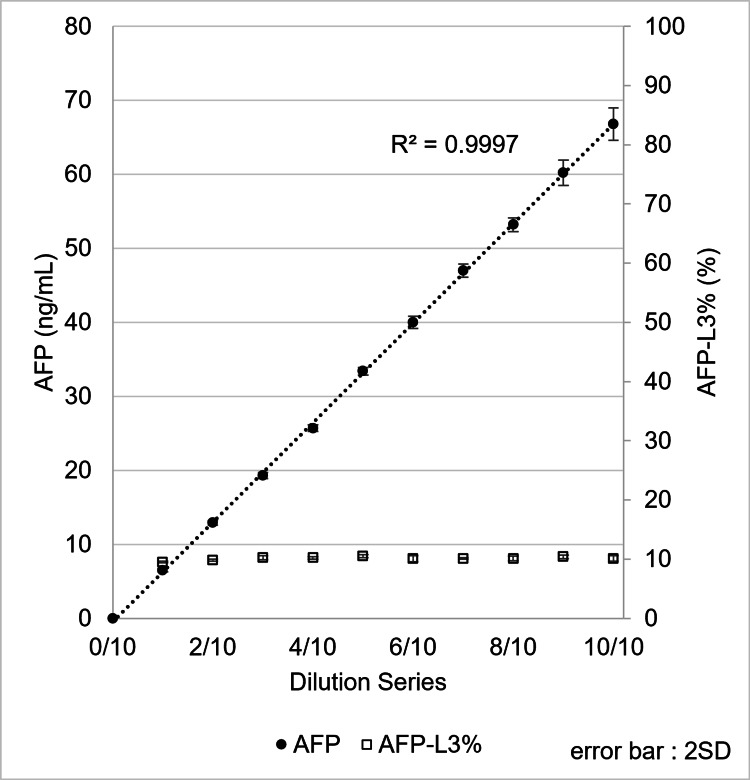
Performance for linearity of AFP, including AFP-L3% at 10%

**Figure 7:**
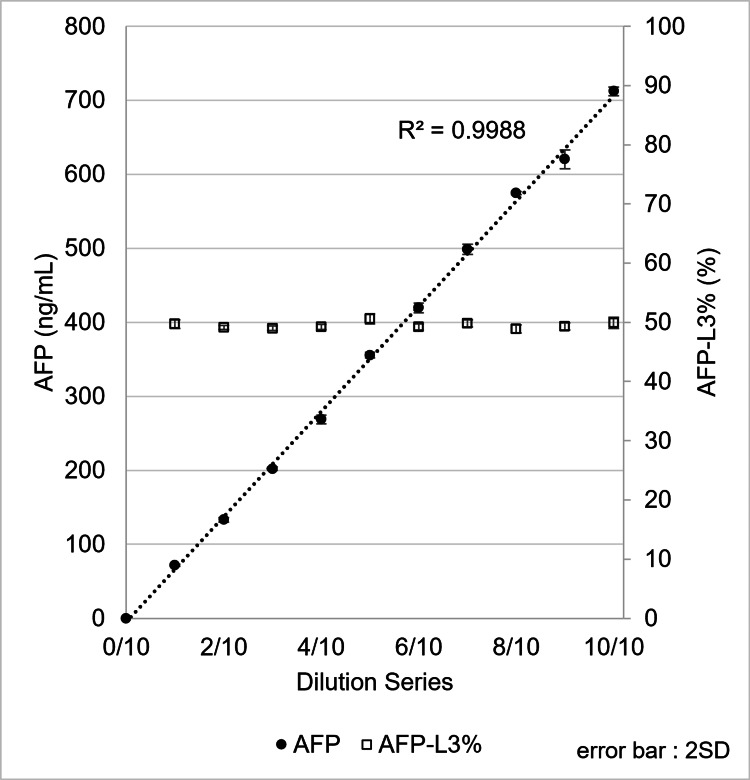
Performance for linearity of AFP, including AFP-L3% at 50%

Two serum samples containing AFP-L3% at approximately 10% and 50% were diluted stepwise with PBS, and the diluted samples were measured using the µTASWako i50. Good linearity of total AFP values was observed at both AFP-L3% levels when plotted against the dilution ratio, and the AFP-L3% remained nearly constant across all dilutions, ranging from 9.5% to 10.4% for the 10% sample and from 48.9% to 50.6% for the 50% sample (Figure 6, Figure 7).

**Figure 8:**
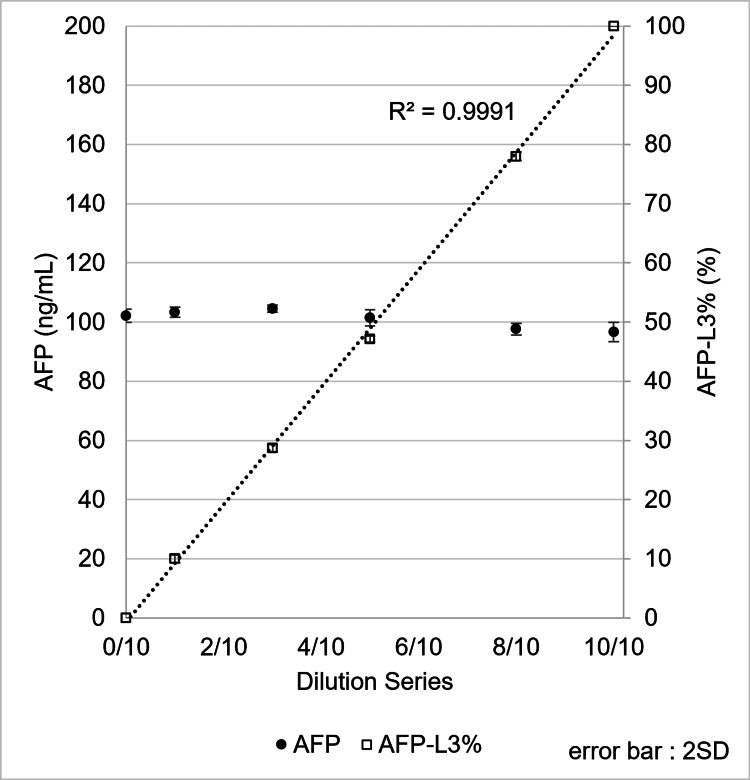
Performance for linearity of AFP-L3%, measured by mixing 100 ng/mL AFP-L1 and 100 ng/mL AFP-L3 at various ratios

Additionally, PBS containing 100 ng/mL AFP-L1 was mixed with PBS containing 100 ng/mL AFP-L3 to generate an AFP-L3% concentration response curve. The mixed samples were measured, and total AFP (L1 + L3) values remained nearly constant. AFP-L3% showed good linearity according to the mixing ratio, with AFP recovery ranging from 96.6% to 104.5%, demonstrating consistency across the tested range (Figure 8). 

PIVKA-II

**Figure 9:**
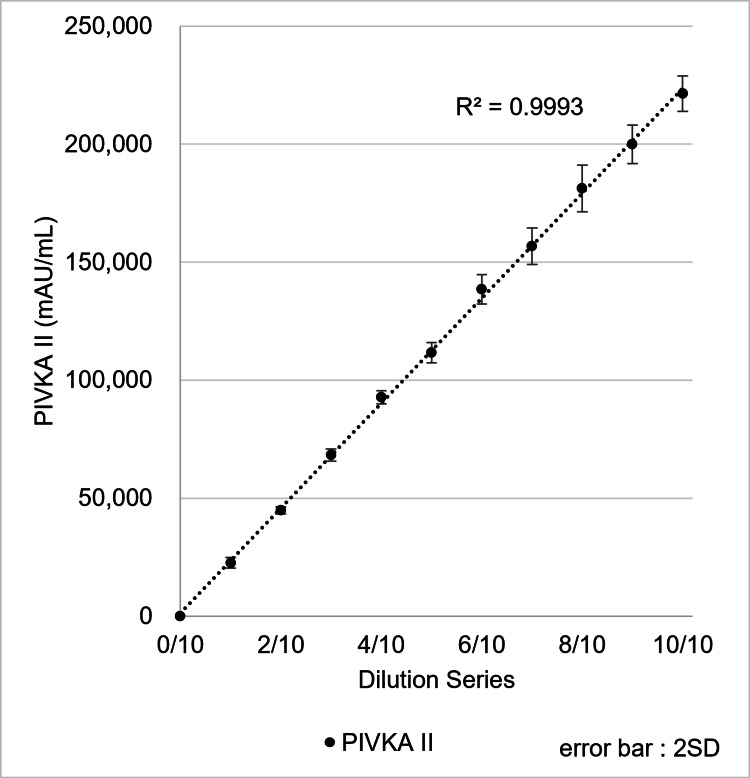
Performance for linearity of PIVKA-II

Good’s buffer, specifically Bis-Tris buffer supplemented with 1% BSA to minimize protein adsorption during measurements, was used as the diluent for PIVKA-II. Good’s buffer containing approximately 220,000 mAU/mL PIVKA-II was serially diluted with PIVKA-II-free Good’s buffer, and the diluted samples were measured using the µTASWako i50. PIVKA-II measurements showed good linearity with respect to the dilution ratio (Figure 9). Based on these results, the measurable range of PIVKA-II could be extended to 200,000 mAU/mL, representing a two-fold improvement over the µTASWako i30 system. For comparison, the US FDA-approved range for the µTASWako i30 system is up to 950 ng/mL DCP (approximately 79,167 mAU/mL PIVKA-II), and the Japan FDA-approved range is 100,000 mAU/mL. Using the automatic sample dilution feature, PIVKA-II concentrations of up to 2,000,000 mAU/mL can be measured with the µTASWako i50.

Assay sensitivity

AFP/AFP-L3%

**Figure 10:**
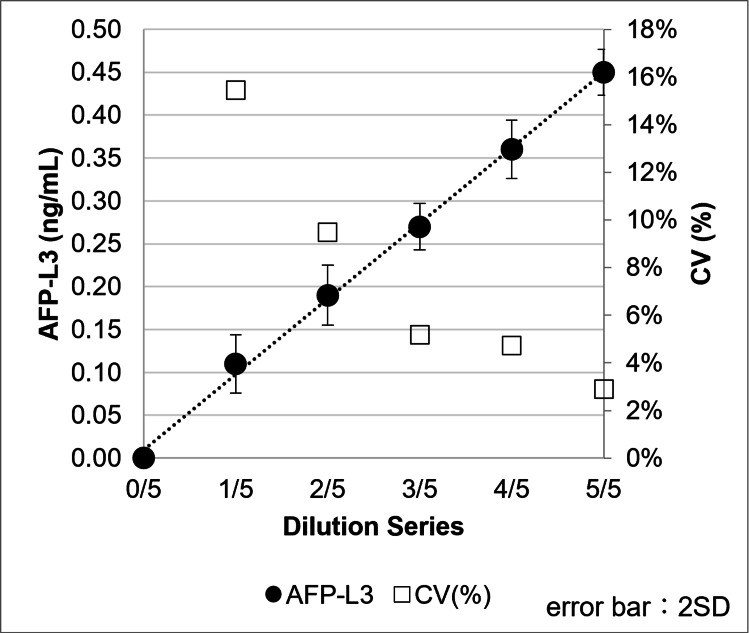
Sensitivity measurements using LOD for AFP-L3 LOD, limit of detection

AFP-free PBS (0 ng/mL) and low-concentration samples (0.1-0.5 ng/mL, serially diluted in PBS) containing AFP-L1 or AFP-L3 were tested using the µTASWako i50 with 21 replicate measurements. The LOD, defined as the concentration at which the ±2 SD range did not overlap with the 0 ng/mL control, was determined to be 0.1 ng/mL or less for both AFP-L1 (data not shown) and AFP-L3 (Figure 10). The LOQ for AFP-L1 was 0.3 ng/mL, based on the criterion that the CV is <5% at the LOQ. These results were comparable to those obtained with the µTASWako i30 system [5].

PIVKA-II

**Figure 11:**
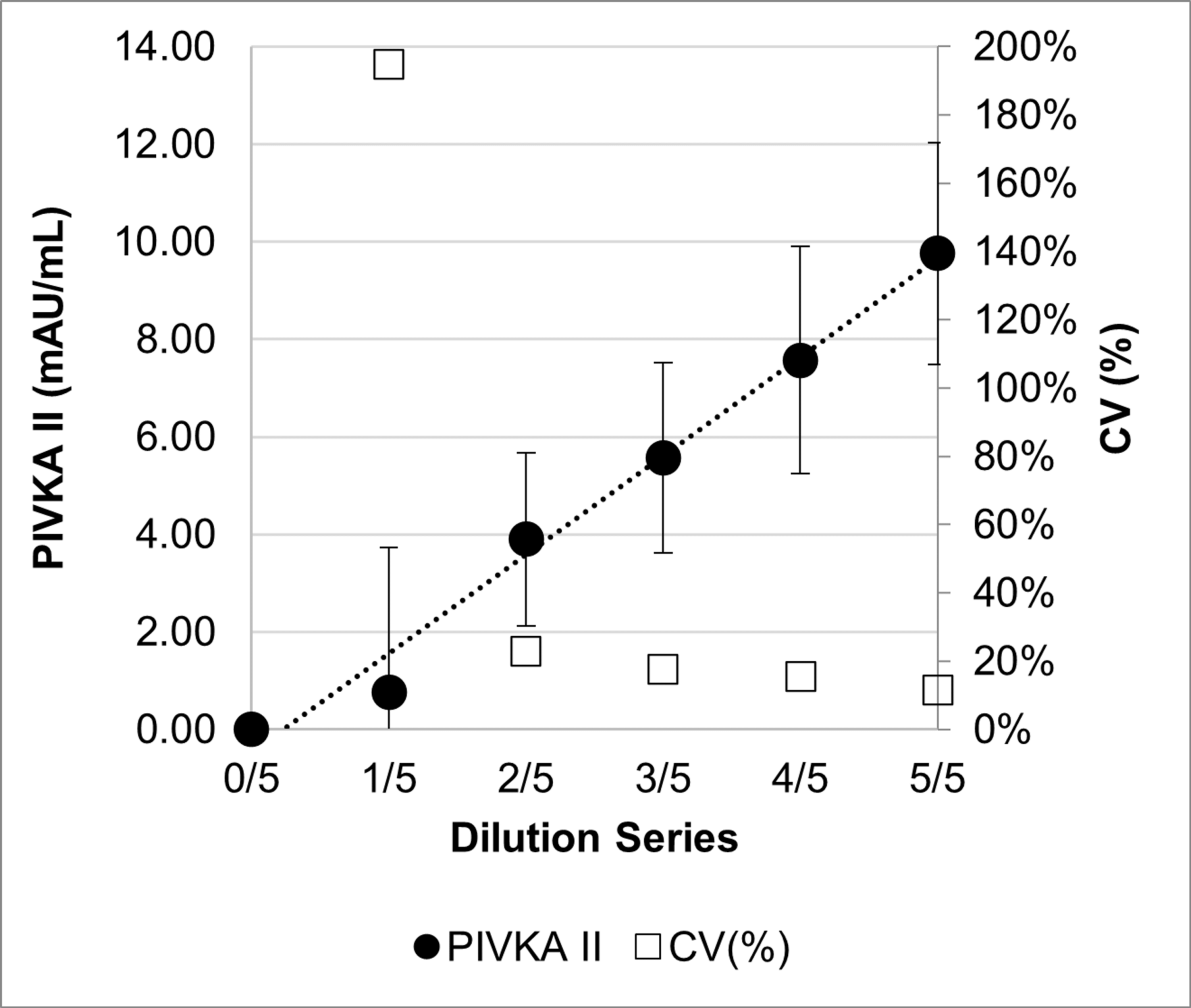
Sensitivity measurements using LOD for PIVKA-II LOD, limit of detection

PIVKA-II-free Good’s buffer (0 mAU/mL, Bis-Tris buffer) and low-concentration samples (2-10 mAU/mL, serially diluted in Good’s buffer as described in the Assay Linearity section) containing PIVKA-II were tested with 21 replicate measurements. The LOD, defined as the concentration at which the ±2 SD range did not overlap with the 0 mAU/mL control, was between 2 and 4 mAU/mL (Figure 11). The LOQ for PIVKA-II was determined to be 10 mAU/mL, based on the criterion that the CV is <10% at the LOQ.

Assay precision

AFP/AFP-L3%

Intra-assay precision was evaluated by calculating the CV for 10 replicate measurements of control samples (Samples 1 and 2) and clinical samples (Samples 3 and 4) with AFP concentrations ranging from 10 to 210 ng/mL and AFP-L3% ranging from 9% to 31%. The CVs for AFP ranged from 1.2% to 2.0%, and for AFP-L3% from 1.0% to 3.5%, indicating precision levels sufficient for diagnostic measurements. Table 2 summarizes these results.

**Table 2:** Precision performance for AFP/AFP-L3% measurements using the µTASWako i50 system

Statistic	Sample 1		Sample 2		Sample 3		Sample 4	
	AFP (ng/mL)	AFP-L3% (%)	AFP (ng/mL)	AFP-L3% (%)	AFP (ng/mL)	AFP-L3% (%)	AFP (ng/mL)	AFP-L3% (%)
n	10	10	10	10	10	10	10	10
Max	53.4	31.7	213.7	20	18.6	15.2	10.9	9.3
Min	49.4	30.8	205.1	19.4	17.6	13.7	10.5	8.9
Range	4	0.9	8.6	0.6	1	1.5	0.4	0.4
Mean	51.4	31.2	210	19.6	18.1	14.5	10.8	9.1
SD	1.05	0.31	2.6	0.19	0.3	0.51	0.14	0.12
CV (%)	2	1	1.2	1	1.7	3.5	1.3	1.3

PIVKA-II

Intra-assay precision (n = 10) for PIVKA-II measurements was evaluated using two control samples (Samples 1 and 2) and one clinical sample (Sample 3). The CVs ranged from 2.8% to 3.2%, demonstrating precision sufficient for diagnostic measurements. Table 3 summarizes these results.

**Table 3:** Precision performance for PIVKA-II measurements using the µTASWako i50 system

Statistic	Sample 1	Sample 2	Sample 3
	PIVKA-II (mAU/mL)	PIVKA-II (mAU/mL)	PIVKA-II (mAU/mL)
n	10	10	10
Max	54	107	1967
Min	49	97	1806
Range	5	10	161
Mean	52	103	1900
SD	1.6	3.3	53.3
CV (%)	3.1	3.2	2.8

Method correlation with µTASWako i30

AFP/AFP-L3%

Clinical specimens from 73 cancer cases and 82 non-cancer cases (155 total) were measured using both the reference method, µTASWako i30, and the µTASWako i50. Regression analysis comparing µTASWako i30 and i50 results for AFP up to 8,000 ng/mL yielded the regression equation y = 0.9817x + 0.089, with a correlation coefficient (r) of 0.9994 (95% CI: 0.9992-0.9996) and a p-value < 0.001. This indicates a very strong correlation and statistical significance. For AFP up to 200 ng/mL, the regression equation was y = 0.9946x - 0.082, with r = 0.9993 (95% CI: 0.9989-0.9995) and p < 0.001, confirming extremely high correlation in the lower range.

**Figure 12:**
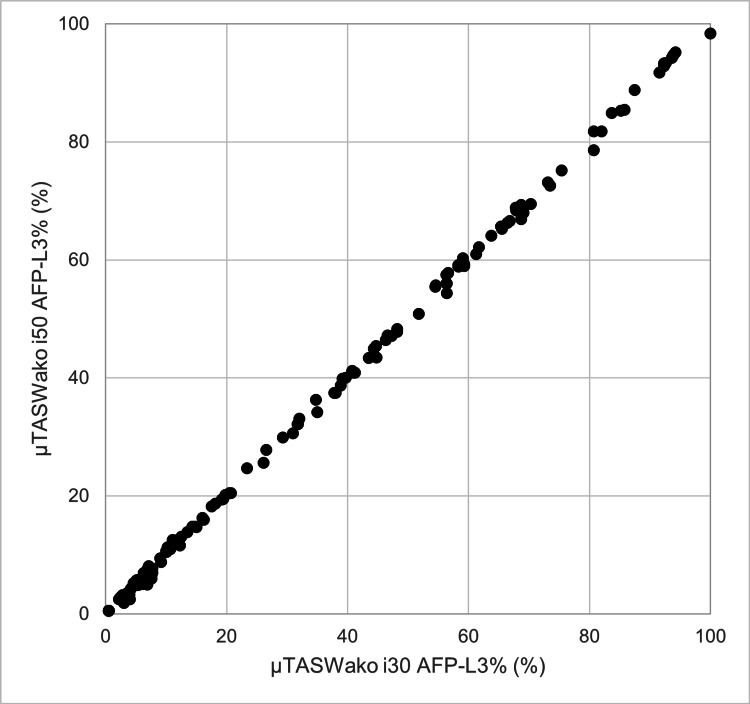
Method comparison of µTASWako i50 AFP-L3% with µTASWako i30 results using 155 patient samples

For AFP-L3%, the regression equation was y = 1.0017x + 0.006, with r = 0.9997 (95% CI: 0.9996-0.9998) and p < 0.001, indicating a strong and statistically significant correlation (Figure 12). Statistical analyses were conducted using JMP^®^ 17.0.0. Using clinically established cutoffs, the agreement rate between the two methods was 100%, demonstrating consistent clinical performance (Table 4).

**Table 4:** Consistency between µTASWako i30 and i50 test results for AFP and AFP-L3%

μTASWako i30/μTASWako i50	AFP ≥10 ng/mL	AFP <10 ng/mL	AFP-L3% ≥10%	AFP-L3% <10%
AFP ≥10 ng/mL	100	0	-	-
AFP <10 ng/mL	0	55	-	-
AFP-L3% ≥10%	-	-	98	0
AFP-L3% <10%	-	-	0	57

PIVKA-II

**Figure 13:**
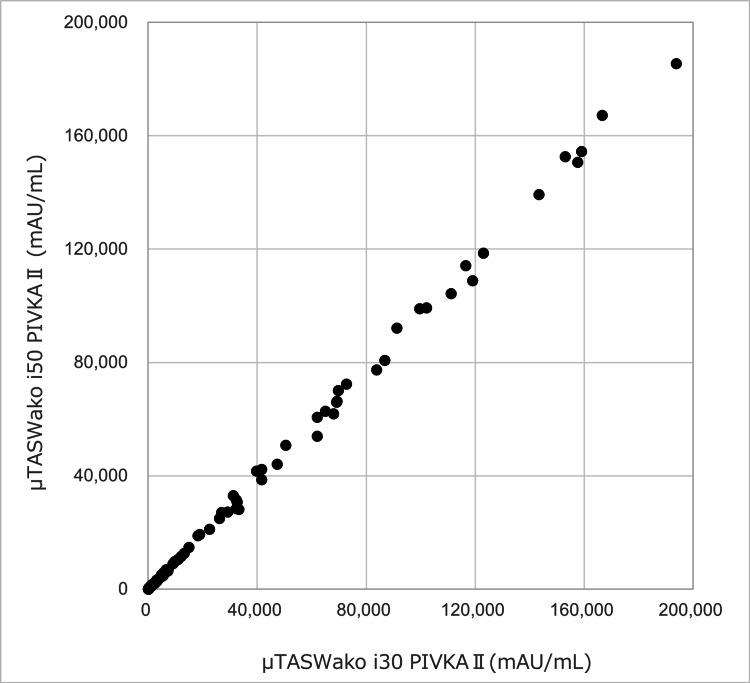
Method comparison of µTASWako i50 PIVKA-II with µTASWako i30 results using 153 patient samples

Clinical specimens from 71 cancer cases and 82 non-cancer cases (153 total) were measured using both µTASWako i30 and µTASWako i50. Regression analysis comparing the two methods for PIVKA-II yielded the regression equation y = 0.9605x + 32.232, with a correlation coefficient (r) of 0.9993 (95% CI: 0.9990-0.9995) and a p-value < 0.001. This strong correlation (r > 0.9) is statistically significant at the alpha level of 0.05 (Figure 13). Statistical analyses were performed using JMP^®^ 17.0.0. Using clinically established cutoffs, the agreement rate between the two methods was 100%, confirming consistent clinical performance (Table 5).

**Table 5:** Consistency between µTASWako i30 and i50 test results for PIVKA-II

μTASWako i30/μTASWako i50	PIVKA-II ≥40 mAU/mL	PIVKA-II <40 mAU/mL
PIVKA-II ≥40 mAU/mL	113	0
PIVKA-II <40 mAU/mL	0	40

Implementation of a warning region for AFP-L2 detection

**Figure 14:**
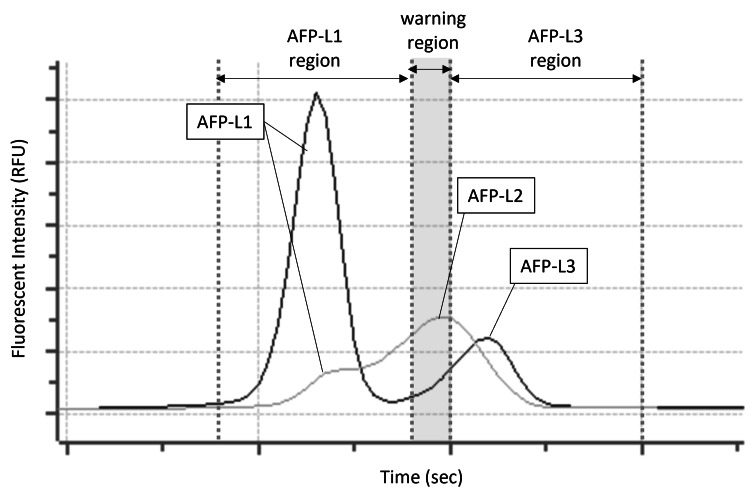
Warning region for the detection of the AFP-L2 peak on the µTASWako i50 system

The µTASWako i50 system incorporates a “warning region” to address potential false-negative AFP-L3% results caused by the presence of the AFP-L2 isoform, which exhibits lower lectin affinity than AFP-L3. This warning region is defined based on the migration time relative to the AFP-L1 peak and allows the system to detect AFP-L2. Operator alerts are triggered when AFP-L2 presence is suspected, enhancing assay reliability and result interpretation (Figure 14).

## Discussion

The increasing prevalence of chronic diseases such as cancer and diabetes has driven a growing demand for rapid and accurate diagnostic tools, stimulating the development of advanced analytical platforms and expanding the application of microfluidic technologies in healthcare. For example, the FREND™ immunoanalytical point-of-care analyzer received FDA approval for SARS-CoV-2 diagnosis [12]. Additionally, Nguyen et al. and Zielke et al. developed electrical cell impedance sensors integrated with microfluidic chips for detecting single cancer cell metastasis [13,14]. A microfluidic system has also been applied to detect low-concentration miRNAs for early-stage breast cancer diagnosis [15]. These platforms rely on separation based on target molecule capture via solid-phase immunoreaction or flow within a microfluidic channel, differing from the µTASWako system, which uses ITP for liquid-phase immunoreaction and bound/free separation, followed by affinity gel electrophoresis to separate analytes with glycan modifications.

In this study, we described the µTASWako i50, developed as a successor to the µTASWako i30 system. The i50 was redesigned to enhance assay throughput and extend the measurement range through automated sample dilution, addressing limitations of the earlier system. These features improve its utility for measuring AFP, AFP-L3%, and PIVKA-II biomarkers associated with HCC, which can exhibit wide concentration ranges in clinical samples. Existing diagnostic platforms for AFP, AFP-L3%, and PIVKA-II, such as Elecsys (Roche Diagnostics, Rotkreuz, Switzerland) and Architect (Abbott Laboratories, Chicago, IL, USA), use chemiluminescence-based immunoassay methods. Elecsys employs electrochemiluminescence immunoassays, whereas Architect uses chemiluminescent immunoassays. Despite technical differences, both methods are well suited for high-throughput routine diagnostics. In contrast, the µTASWako i50 system employs a unique microfluidic-based ITP and CGE approach, enabling simultaneous quantitative and qualitative analysis of glycosylation variants such as AFP-L3. Unlike some platforms requiring separate measurements for AFP and AFP-L3 to calculate AFP-L3%, the µTASWako i50 allows simultaneous quantification of total AFP and AFP-L3%, providing a streamlined approach for glycosylation biomarker analysis. Combined with automated sample dilution and an expanded measurement range, these features position the i50 as a complementary tool, particularly where advanced glycoprotein biomarker analysis is required.

The analytical performance of the i50 system, including linearity, sensitivity, and reproducibility, was comparable to the i30 system. Testing of clinical specimens demonstrated a strong correlation between i30 and i50 results, supporting the i50’s suitability for laboratories requiring higher throughput and greater automation. Moreover, the implementation of a “warning region” in the i50 system addresses a limitation observed with the predecessor regarding AFP-L3% detection in the presence of the AFP-L2 isoform, which has lower lectin affinity. By enabling detection of AFP-L2 and providing operator alerts, this feature may reduce missed detection of lectin-affinity variants and improve assay reliability relative to the i30 system.

Glycosylation pattern alterations, such as those observed in AFP-L3, are increasingly recognized as clinically relevant cancer biomarkers. Nevertheless, their routine diagnostic adoption remains limited due to assay complexity and cost. Malignant transformation often results in heterogeneous glycan modifications [16], highlighting the advantage of separation methods such as lectin affinity electrophoresis used in the µTASWako i50 for resolving and quantifying these variants. The i50 system’s ability to differentiate AFP isoforms, including AFP-L1, AFP-L2, and AFP-L3, demonstrates its capability for automated and reproducible glycoprotein biomarker measurement. Although this study focuses on liver cancer biomarkers, this technology can also be applied to glycosylation-related biomarkers in other diseases, warranting further exploration. Previous studies have shown its potential for expanding biomarker discovery, including glycation-associated isoforms such as α2,3-linked sialyl N-glycan-carrying prostate-specific antigen (PSA) [17] and core-type fucosylated PSA [18]. The discovery of new glycosylation-related biomarkers may broaden the clinical applications of the µTASWako i50, which demonstrates high sensitivity and precision for detecting molecular variants.

Simultaneous analysis of glycosylation patterns, whether on the same protein or across different proteins, has the potential to provide complementary insights into disease biology, enhancing biomarker clinical utility. In oncology, integrating multiple biomarkers has been shown to improve diagnostic and prognostic accuracy. For example, combining AFP, AFP-L3%, and PIVKA-II improves HCC diagnostic performance, even at lower total AFP levels (<20 ng/mL) [19]. Similarly, joint monitoring of HE4 and CA125 improves sensitivity and specificity for epithelial ovarian cancer diagnosis [20]. These examples illustrate the potential of combining glycan structure analyses to yield clinically meaningful results. Combined assessment of sialylation and core-type fucosylation of PSA has shown promising diagnostic performance in distinguishing high-grade prostate cancer [21]. The integration of advanced separation technologies further highlights the µTASWako i50’s potential to address unmet clinical and research needs in glycan structural analysis.

Beyond HCC biomarker research, the µTASWako i50 is well-positioned to contribute to broader biomarker discovery and cancer-related diagnostic technologies. By supporting translational research and enabling improved detection and monitoring of complex diseases, the system provides a robust framework for advancing diagnostic and prognostic applications.

However, several limitations of this study should be acknowledged. First, the study was conducted as a retrospective analysis using a relatively limited dataset of residual clinical samples from a single institution. This design enabled an initial and efficient evaluation of the analytical performance of the µTASWako i50 system; however, it inherently limits the diversity and representativeness of the study population. Consequently, the generalizability of these findings may be restricted, and further validation across larger and more diverse patient cohorts is warranted to confirm applicability in broader healthcare settings. Second, this study primarily focused on the analytical performance of the µTASWako i50 system in comparison to its predecessor, the µTASWako i30. While correlation and precision metrics were highly promising, additional clinical investigations are needed to evaluate the real-world impact of improvements such as enhanced sensitivity, increased throughput, and automation on patient care and diagnostic workflows. Although the analytical advantages of the µTASWako i50 system were robustly demonstrated in this study, their broader clinical benefits remain to be validated in future prospective studies. Third, while detailed biochemical specifications for AFP have been previously reported, certain assay components for PIVKA-II, particularly specific antibody clone identities, are proprietary and not publicly disclosed, representing a standard limitation of commercially available diagnostic systems. Although the AFP and PIVKA-II assays share the same immunoassay format and antibody type, minor analyte-specific differences in buffer composition exist. Finally, this study evaluated only three biomarkers (AFP, AFP-L3%, and PIVKA-II) associated with HCC. Additional assessments are needed to determine the system’s performance in detecting other biomarkers or diseases.

Future research should evaluate the system’s broader applicability, particularly for detecting biomarkers related to diseases beyond HCC and those with diverse post-translational modifications, such as glycosylation and phosphorylation. Diseases such as pancreatic and ovarian cancer, where glycosylation-related biomarkers have shown significant clinical promise, represent key areas for further exploration. For HCC, a large-scale, prospective, multicenter cohort study involving several hundred to over one thousand patients with liver cirrhosis across different etiologies could provide robust validation of the system’s clinical utility. These studies could also explore the system’s potential for multiplexed assays and point-of-care applications, paving the way for integration into personalized medicine. Achieving these advancements will require close collaboration between technology developers and clinical researchers to improve early disease detection, enhance molecular characterization, and ultimately advance patient care.

## Conclusions

The µTASWako i50 system represents a significant advancement in microfluidic immunoassay technology, offering enhanced utility for clinical diagnostic applications. By improving throughput, expanding the measurement range through automated sample dilution, and maintaining high sensitivity, linearity, and reproducibility, the system enables efficient and accurate quantification of key HCC biomarkers, including AFP, AFP-L3%, and PIVKA-II. The redesigned microfluidic chip and enhanced affinity electrophoresis capabilities provide improved resolution and specificity for glycoprotein isoforms, ensuring reliability in clinical operations.

Future studies should focus on validating the system across diverse healthcare settings and conducting independent, large-scale evaluations to establish its applicability in broader patient populations and more complex disease scenarios. Additionally, the system’s capacity for multiplexed assays and its compatibility with point-of-care applications represent significant opportunities to enhance diagnostic workflows and support personalized medicine.
